# BK_Ca_ Channel Activation Increases Mitochondrial Supercomplexes and Improves Cardiac Recovery in a Large Animal Model of Myocardial Stunning Following Cardioplegic Arrest and Cardiopulmonary Bypass

**DOI:** 10.3390/antiox15101366

**Published:** 2026-10-21

**Authors:** Wenzhuo Ma, Joseph Owusu-Sarfo, Zhiqi Zhang, Scarlett M. Silvia, Brenda Cordeiro, Lauren F. Bazinet, Thomas Mancini, Benison C. Aguocha, Ahmed Aboulgheit, Jin O-Uchi, Bong Sook Jhun, Afshin Ehsan, Gaurav Choudhary, Frank W. Sellke, Jun Feng, Martin Kolodziejczack, Dmitry Terentyev, Neel R. Sodha, Richard T. Clements

**Affiliations:** 1 Department of Biomedical and Pharmaceutical Sciences, College of Pharmacy, University of Rhode Island, Kingston, RI 02881, USA; wenzhuo_ma@uri.edu (W.M.); jowusu-sarfo@uri.edu (J.O.-S.); scarhuck@gmail.com (S.M.S.); lauren.bazinet@uri.edu (L.F.B.); benison@uri.edu (B.C.A.); 2 Department of Surgery, Alpert Medical School, Brown University, Providence, RI 02912, USA; zhiqi_zhang@brown.edu (Z.Z.); bcordeiro925@gmail.com (B.C.); mancinit93@gmail.com (T.M.); aligheit89@gmail.com (A.A.); afshin.ehsan@bswhealth.org (A.E.); fsellke@lifespan.org (F.W.S.); mkolodziejczak1@brownhealth.org (M.K.); 3 Ocean State Research Institute and VA Providence Health Care System, Providence, RI 02908, USA; gaurav_choudhary@brown.edu; 4 Hypertension and Kidney Research Center, Department of Molecular Pharmacology and Physiology, Heart Institute, Morsani College of Medicine, University of South Florida, Tampa, FL 33602, USA; jouchi@usf.edu (J.O.-U.); bjhun@usf.edu (B.S.J.); 5 Department of Surgery, Heart Institute, Morsani College of Medicine, University of South Florida, Tampa, FL 33602, USA; fengj@usf.edu; 6 Department of Physiology and Cell Biology, The Ohio State University, Columbus, OH 43210, USA; dmitry.terentyev@osumc.edu

**Keywords:** ischemia, BK_Ca_, Slo1, supercomplex, respirasome, mallotoxin, potassium, cardioprotection

## Abstract

Cardiac surgery or transplant using cardioplegic arrest and cardiopulmonary bypass (CP/CPB) subjects the heart to mild reversible ischemic injury that results in pronounced myocardial stunning. Mitochondrial large conductance calcium-activated K^+^ channels (BK_Ca_) in cardiomyocytes are potently cardioprotective and beneficially modulate respiration and ROS post-I/R injury. However, how BK_Ca_-dependent K^+^ flux regulates ROS and respiration is unclear. We evaluated the BK_Ca_ activator rottlerin in a preclinical large animal pig CP/CPB model of myocardial stunning and evaluated BK_Ca_-dependent cardioprotective mechanisms in vitro and in vivo. BK_Ca_ activation with the small molecule rottlerin significantly improved LV cardiac functional recovery and contractility at 1 h reperfusion. TEM showed larger mitochondria in the rottlerin group consistent with K^+^ influx. We also found that pigs with rottlerin treatment had increased electron transport chain supercomplexes (mSCs), which are thought to improve respiratory efficiency and reduce ROS. Consistent with increased mSC, rottlerin reduced ROS-dependent protein modification in CP/CPB pigs. In complementary studies, rottlerin and/or adenoviral overexpression of a mutant mito-BK_Ca_ increased respiration, altered mitochondrial morphology, increased mSC assembly, and significantly reduced ROS following in vitro CP/reoxygenation, similar to CP/CPB pigs. In summary, mito-BK_Ca_ activation increases mSCs and respiration while reducing ROS, suggesting that mSC-dependent improved respiratory efficiency promotes robust cardioprotection.

## 1. Introduction

Myocardial stunning is defined by reduced cardiac contractile function in viable cardiac tissue due to reversible ischemic injury. It is an important clinical problem resulting in depressed cardiac output and tissue perfusion. Global myocardial stunning can result from ischemic insults associated with cardiac surgery using cardioplegic arrest and cardiopulmonary bypass (CP/CPB) or cardiac transplant [1,2,3]. Regional myocardial stunning is present clinically following acute myocardial infarction (MI) and early revascularization therapy, off-pump coronary artery bypass grafting (CABG), and percutaneous coronary interventions such as angioplasty and stent placement [4]. Although stunning resolves in the majority of patients, severe stunning in ~10% of CP/CPB cases can persist and require inotropic and/or mechanical circulatory support, prolongs hospital stays, increases the chance of mortality, and greatly increases economic burden [5,6]. Therefore, improved cardioprotection strategies are needed in cases of surgical cardiac arrest and transplant.

Mitochondrial K^+^ channels, including ATP-sensitive K^+^ channels (KATP), small conductance Ca^2+^-activated K^+^ (SK) channels and large conductance Ca^2+^-activated K^+^ (BK_Ca_) channels, have long been recognized as having potent cardioprotective activities associated with improved mitochondrial function [7,8]. Importantly, unlike KATP and SK channels, BK_Ca_ channels are absent in sarcolemma of cardiomyocytes while being solely expressed in the inner mitochondrial membrane, mitigating arrythmia concerns [9,10,11,12]. Cardiomyocytes exclusively express a c-terminal “DEC” splice variant found only in the IMM [12]. BK_Ca_ channels are potent regulators of mitochondrial volume [13]. Activation of BK_Ca_ channels is associated with reductions in reactive oxygen species (ROS) production, reduced mitochondrial permeability transition pore (mPTP) opening, modulation of mitochondrial membrane potential and improved respiration [14,15,16,17,18,19,20]. In irreversible I/R injury rodent models, BK_Ca_ activation or overexpression of BK_Ca_ channels is associated with improvements in viability, reductions in infarct size, and improved cardiac function [12,14,15,16,19] Conversely, cardiomyocyte specific BK_Ca_ depletion exacerbates I/R injury [20].

Our group has previously demonstrated that cardioplegia solutions supplemented with the BK_Ca_ activator rottlerin improve cardiac recovery 30 min post cold cardioplegic arrest in a myocardial stunning model in isolated perfused rodent hearts [17,18]. Rottlerin is a highly potent BK_Ca_ small-molecule activator, with nM concentrations capable of shifting the voltage-dependent activation of the channel approximately −70–100 mV, allowing for more frequent channel opening in the highly hyperpolarized mitochondrial membrane [21,22]. Rottlerin-induced cardioprotection in our CP/R-induced myocardial stunning models was BK_Ca_-specific as cardiac functional improvements were absent in BK_Ca_ KO mice [17]. We also found in cell models of hypoxic cardioplegia and reperfusion that rottlerin blocked CP-induced ROS generation and mitigated reductions in ΔΨm, suggestive of improved mitochondrial function and preserved respiration [17].

Despite the well-recognized mitochondrial functional improvements associated with K^+^-channel activation post ischemia, downstream mechanisms of how K^+^ flux improves ROS and modulates respiration is unclear. We hypothesized that mitochondrial K^+^ flux and subsequent regulation of mitochondrial volume may modulate mitochondrial structure to promote and/or preserve mitochondrial electron transport chain supercomplexes (mSCs). mSCs consist of ETC CI, III and IV in physical association and are thought to improve mitochondrial respiratory efficiency [23,24]. Importantly, mSC-mediated respiration is associated with increased respiratory efficiency linked to reduced mitochondrial ROS generation and consequently less oxidative damage.

To determine the efficacy of BK_Ca_-dependent cardioprotection, we employed a highly translational, clinically relevant large animal pig CP/CPB model using BK_Ca_ activator-supplemented cardioplegia. Pig hearts were arrested with or without rottlerin-supplemented cardioplegic solution and put on cardiopulmonary bypass for 1 h, followed by weaning and reperfusion for 1 h. Rottlerin-dependent changes in mitochondrial size, supercomplex assembly, and ROS-dependent protein modification were assessed. We further assessed the effects of rottlerin and/or mitochondrial-specific BK_Ca_ overexpression on mitochondrial morphology, respiration and ROS generation in an in vitro cell model of CP/reoxygenation (CP/ReO2). To our knowledge, this is the first study demonstrating robust BK_Ca_-dependent cardioprotection in a large animal model of myocardial stunning and reversible ischemic injury. Furthermore, our results demonstrate that genetic BK_Ca_ activation robustly promotes mSC assembly, suggesting that this may be a central mechanism associated with BK_Ca_-dependent improvements in mitochondrial function and reduced ROS-mediating cardioprotection.

## 2. Materials and Methods

**Animals and CP/CPB:** Pigs were ~50 kg male and female Yorkshire swine (sham (3M, 2F), CP/CPB (4M, 3F) and CP/CPB + Rott (4M, 2F)). Sample size (n = 7) was determined based on preliminary studies in rodent isolated hearts using hemodynamic function (LVDevP) and expecting a 25±15% difference of means, alpha of 0.05 and power of 0.8 using *t*-tests. Pigs were ordered to the facility in groups of 4–5 pigs of single sex and similar age and socially housed when possible. Pigs were acclimatized for 1 week and used within 2–3 weeks of arrival. Pigs were assigned to CP/CPB or CP/CPB + rott groups in an alternating pattern the day prior to procedure with randomized initial assignment. Sham pigs were assigned on days a trained cardiothoracic surgeon was unavailable.

Animals were initially sedated with telazol (5 mg/kg)/xylazine (2 mg/kg), intubated and then maintained on ~3% isoflurane. Fluid support (saline) was given at a rate of 10 mL/kg/h supplemented with 0.15 µg/kg/min phenylephrine to combat anesthesia-induced vasodilation. A cut down was performed on the right groin/hind limb to expose the femoral artery, and a Millar pressure catheter (Pearland, TX, USA) was inserted and advanced to the aortic arch. A sternotomy was performed and a pericardial cradle constructed. A double-stage 29 fr venous cannula was inserted into the inferior vena cava via the right atrial appendage and secured with a purse string. A 12 fr arterial cannula was inserted in the ascending aorta ~7–8 cm above the aortic root. A cardioplegia delivery cannula was inserted in the aorta ~3–4 cm above the aortic root. A Millar seven-segment pressure volume catheter was inserted in the apex of the LV and secured with a u-stitch. The bypass circuit with a reservoir and oxygenator (Cobe, Arvada, CO, USA) was primed with 2 L of plasmalyte solution. The cardiopulmonary bypass pump comprised a Cobe roller pump system with a main perfusion circuit, cardioplegia circuit, suction, and RV vent.

Pigs were heparinized (15,000 U) and hypothermic cardiopulmonary bypass (32 °C) was initiated with flow rates ~2–3 L/min to maintain perfusion pressure at >50 mmHg. A cross clamp was placed between the cardioplegia and arterial cannulas and cardiac arrest was initiated by infusion of 300 mL of cold (4 °C) blood/cardioplegia mix (4:1 mix). Induction cardioplegia solution consisted of 75.6 mM tromethamine, 72.3 mM glucose, 39.5 mM mannitol, 20.3 mM MgSO4, and 100 mM KCl made in Plasma-Lyte A. Maintenance doses of 150 mL delivered at 20 and 40 min were similar to induction doses but contained 40 mM KCl. In the CP/CPB+Rott group, rottlerin (500 nM, Tocris: Bio-Techne/R&D Systems, Minneapolis, MN, USA) was included in the cardioplegia solution, and an equivalent concentration of DMSO (vehicle 1:20,000) was added in the CP/CPB group. To avoid pulmonary atelectasis, pigs remained ventilated with low frequency (~5/min) and tidal volume (100–150 mL) during bypass. During bypass, activated clotting time (ACT) was monitored and additional heparin added as needed. Blood gases and electrolytes including Ca^2+^ and K^+^ were monitored throughout bypass with a blood gas analyzer (Optimedical), and K^+^ levels were managed with gas flow rate manipulation and/or bicarbonate. Hearts were packed with sterile saline slush during arrest.

The bypass circuit was rewarmed to approximately 37 °C and cardiac arrest terminated via removal of the aortic cross clamp. Contractile and electrical activity were allowed to recover spontaneously, with defibrillation used if necessary. Hearts were allowed to rest for ~5 min while supported by bypass. Following 5 min of rest, hearts were weaned from bypass by slowly reducing flow rate and returning volume to the pig over ~5 min, monitoring RV volume status. Ca^2+^, lidocaine, and defibrillation were given as needed for arrhythmia management during the weaning process and early reperfusion. No inotropic drugs or other support were provided to animals during reperfusion or weaning to allow for non-confounding interpretation of rottlerin’s effects on contractility. Control (sham) animals were subjected to sternotomy, aortic and LV catheter placements and were monitored for 2 h. IVC occlusions were performed at baseline (pre-bypass) and at 1 and 2 h. IVC occlusions were performed in CP/CPB and CP/CPB + rott groups at baseline and at 1 h reperfusion. One pig in the CP/CPB group was excluded from end-systolic elastance calculations due to poor-quality volume traces prohibiting ESPVR analysis. Individuals responsible for analyzing hemodynamic and EM data were blinded to pig group identity by the PI (Clements).

**Electron microscopy:** Small pieces of LV (1 mm × 1 mm × 1 mm) were placed in EM fixative (2.5% glutaraldehyde, 2% paraformaldehyde, 0.15 M sodium cacodylate buffer, 2 mM CaCl_2_) at 4 °C. Tissue was stained with filtered thiocarbohydrazide solution and then stained/contrasted with 2% osmium tetroxide for 30 min. Following dehydration, samples were embedded in Epon resin. Blocks were sectioned and imaged according to standard methods. Three sham, four CP/CPB and three CP/CPB + rott pigs were randomly selected for EM analysis.

**SDS-PAGE and immunoblot:** Mitochondrial ETC protein content and overexpression of mBKQ were visualized using SDS-PAGE and immunoblot with an OXPHOS antibody cocktail (1:1000; Abcam, Cambridge, UK: ab110413), tropomyosin (1:1000; Sigma-Aldrich, St. Louis, MO, USA: T2780), BKca (1:200; Alomone Labs: #APC-021), Flag (1:1000, Sigma-Aldrich: F1804), and beta-actin (1:1000, Cell Signaling Technology, Danvers, MA, USA: 4967). OxyBlot Protein Oxidation Detection Kit (Sigma-Aldrich: S7150) was used to detect protein carbonyl modification in isolated mitochondrial fractions according to the manufacturer’s protocol. Briefly, 20 µg mitochondrial protein lysate was collected from pig LV tissue, and carbonyl groups were derivatized with 2,4-dinitrophenylhydrazine (DNPH) for 15 min at room temperature. A parallel aliquot from each sample was treated with the derivatization control solution as a negative control. Reactions were neutralized, separated by SDS-PAGE, transferred, and blotted with anti-DNP primary antibody, followed by HRP-conjugated secondary antibody.

**Mitochondrial isolation and BN-PAGE**: Pig LV tissue (~100 mg) mitochondria were isolated via tissue disruption with Dounce homogenization in Mitochondria Isolation Buffer (MIB: 0.28 M sucrose, 10 mM HEPES and 2 mM EDTA, PH 7.4) containing protease and phosphatase inhibitor cocktails (Sigma-Aldrich) and using standard differential centrifugation techniques (2000 g, 5 min, then pellet mitochondria at 13,000× *g*, 15 min at 4 °C). Mitochondrial supercomplex assembly was assessed via Blue-Native PAGE and immunoblot with antibodies specific to complex I (NDUFA9; 1:1000; Abcam: 14713), complex IV (Cox IV; 1:1000; Abcam: 14746) and complex III (UQCRFS1; 1:1000; Abcam: 14744).

**Seahorse Assay**: H9c2 cells were cultured in Agilent Seahorse XFe96/XF Pro Cell Culture Microplates (Agilent Technologies, Santa Clara, CA, USA) at 1 × 10^5^ cells/well After 24 h, H9c2 cell medium was replaced with DMEM containing 1% FBS to initiate a 7-day differentiation protocol resulting in a cardiac-like phenotype. On day 5, cells were infected with adenovirus encoding either mCherry (control) or mitochondrial-targeted BK_Ca_ with an mCherry tag (mBKQ). After 48 h, cell medium was switched to cold St. Thomas II solution (110 mM NaCl, 16 mM KCl, 16 mM MgCl_2_, 1.5 mM CaCl_2_, 10 mM NaHCO_3_) and the plate containing the hypoxia group was placed in a hypoxia chamber which was purged with 5% CO_2_, 95% N_2_ at 15 L/min for 5 min. The chamber was maintained at room temperature for 3 h without disturbance. For the H9c2 rottlerin treatment assay, cells were treated with either rottlerin (500 nM) or vehicle (0.05% DMSO) in St. Thomas II solution and placed in the hypoxia chamber as described previously. Normoxia group plate cell medium was replaced with either rottlerin or vehicle in DMEM and incubated for 3 h at 37 °C. After 3 h, both control and hypoxia plates were switched to phenol and bicarbonate-free DMEM with 1 mM pyruvate, 2 mM glutamine, and 10 mM glucose (Agilent) and maintained under normoxia at 37 °C for 30 min. H9c2 cell respiration was measured by using the Agilent Seahorse XFe96 Analyzer according to the manufacturer’s protocol. Briefly, a hydrated sensor cartridge was loaded with 25 µL of the following compounds to achieve the final listed concentration: oligomycin (3 µM, Thermo Fisher Scientific, Waltham, MA, USA), carbonyl cyanide-p-trifluoromethoxyphenylhydrazone (FCCP) (1.5 µM, Sigma-Aldrich, St. Louis, MO, USA), and a mix of rotenone (2 µM, Millipore, Burlington, MA, USA) and antimycin A (2 µM, Thermo Fisher Scientific, Waltham, MA). After performing the assay, H9c2 cells were stained with Hoechst 33342 (1 µg/mL, Thermo Fisher Scientific, Waltham, MA, USA) for cell number normalization.

**Mitochondrial Morphology:** H9c2 cells stably expressing a mitochondrial matrix-targeted red fluorescent protein (mito-RFP-H9c2) were used for live-cell imaging and quantitative analysis of mitochondrial morphology according to [25]. Cells were plated in glass-bottom tissue culture dishes and infected with Adv-GFP or Adv-mBKQ-IRES-GFP for 48 h and exposed to our in vitro CP/ReO_2_ protocol; after 30 min, the culture medium was replaced with Tyrodes buffer (137 mM NaCl, 2.68 mM KCl, 1.0 mM MgCl_2_, 11.90 mM NaHCO_3_, 0.42 mM NaH_2_PO_4_, 1.8 mM CaCl_2_, 5.6 mM glucose, pH 7.4), and focused z-stack projection images were collected on a Nikon fluorescent microscope at room temperature. Quantitative analyses of mitochondrial morphology were performed using ImageJ-1.5g as previously described [25]. Focused z-stack projection images were processed using a convolution filter and converted to binary masks. Individual mitochondria were analyzed for form factor (FF; reciprocal of circularity) and aspect ratio (AR; major axis/minor axis). A higher AR and FF indicate elongated tubular mitochondria and increased mitochondrial length and complexity, respectively.

**HyPer7 ROS Assay:** H9c2 cells (ATCC, Manassas, VA, USA) were seeded in Corning™ 384-Well Cell Culture-Treated Clear Flat-Bottom Microplates (Fisher Scientific, Waltham, MA, USA). Cells were subsequently infected with adenovirus encoding mitochondrial matrix-targeted HyPer7 [26], with or without mitochondrial-targeted BK_Ca_ (mBKQ). After 48 h, cells were washed three times with pre-warmed Tyrode’s solution and then the cell plate was placed in the Cytation 5 Imager (Agilent Technologies) with temperature and gas controls set to 21% O_2_, 5% CO_2_, and 37 °C. Baseline fluorescence images were acquired every 15 min for 45 min. Following baseline measurements, cells were switched to cold St. Thomas II solution to mimic ischemic conditions. Instrument conditions were adjusted to <1% O_2_ and 5% CO_2_. Fluorescence images were acquired every hour for 4 h. Following the ischemic period, cells were returned to Tyrode’s solution, and instrument conditions were restored to 21% O_2_, 5% CO_2_, and 37 °C for the reoxygenation phase. Fluorescence images were collected every 15 min for 1 h. At the end of the assay, DTT (Bio-Rad Laboratories, Hercules, CA, USA) was added to a final concentration of 5 mM followed by H_2_O_2_ (Fisher Scientific, Waltham MA, USA) at a final concentration of 600 µM to determine the minimum and maximum oxidation states of HyPer7, respectively. ROS were quantified by using the HyPer7 fluorescence ratio F488/F400 and then normalized to the minimum (DTT) and maximum (H_2_O_2_). Cells were automatically detected using Biotek Gen5 software based on 488ex/535 em fluorescence intensity, calculated individual 488/400 ex ratios, and mCherry fluorescence intensity. Individual cells were averaged/well and only mCherry-positive cells were included in calculations in the mBKQ groups.

**Statistics:** All statistical analyses were performed with Graphpad Prism 11.0.0. One-way ANOVA with Student’s–Neuman–Keuls was used for 3-group analysis or two-way ANOVA with Holm–Sidak, as indicated in the legends.

## 3. Results

**Figure 1:**
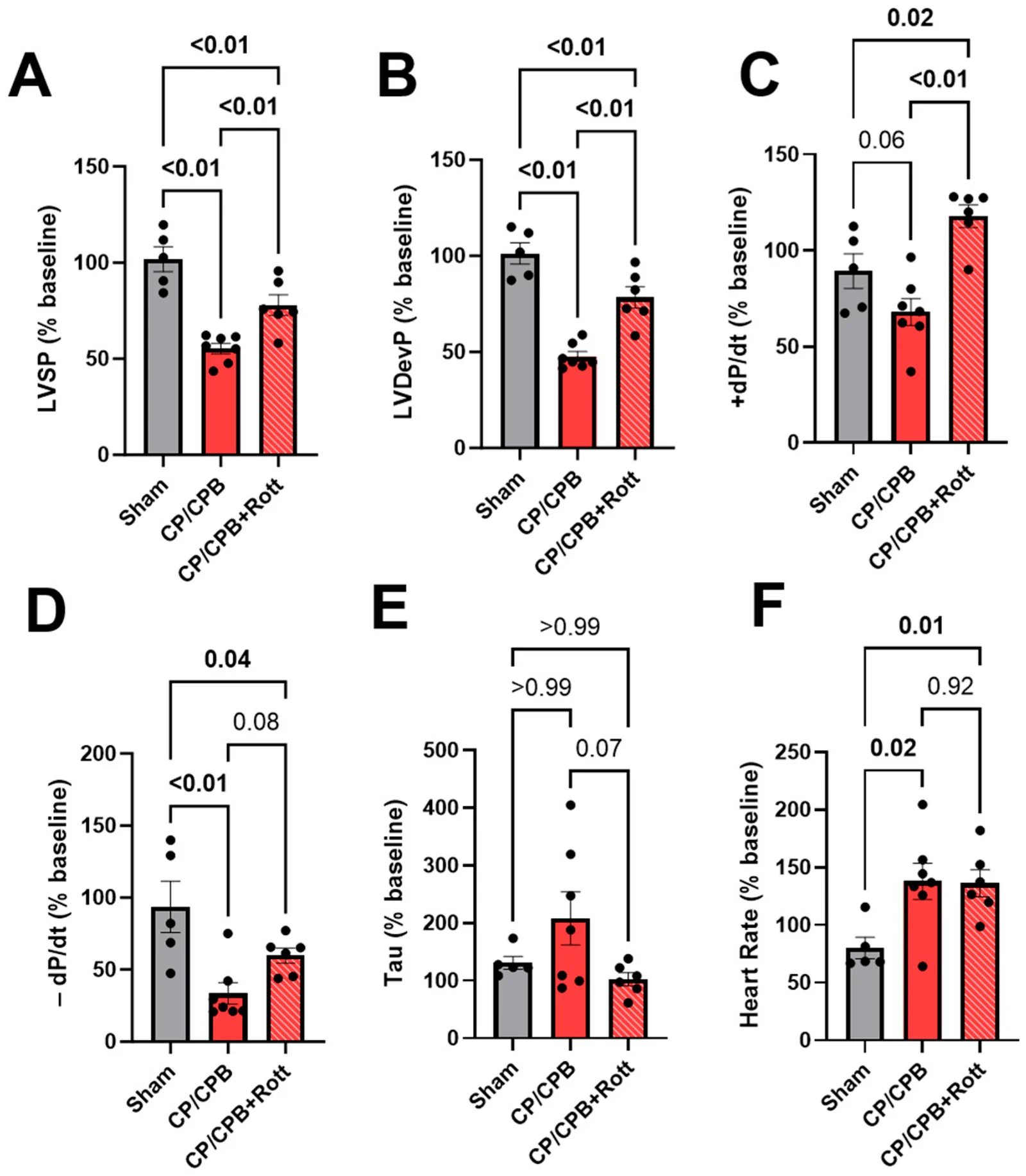
***Rottlerin improves cardiac functional recovery post CP/CPB.*** Hemodynamic parameters at 60 min reperfusion post bypass or 2 h after sham surgery. (**A**) LVSP, (**B**) LVDevP, (**C**) +dP/dt, (**D**) −dP/dt, (**E**) Tau, and (**F**) heart rate. Data are expressed as % change from baseline pre-bypass values. Sham n = 5, CP/CPB n = 7, and CP/CPB + Rott n = 6. *p* as indicated; one-way ANOVA with SNK.

***Rottlerin improves cardiac contractility and cardiac function post-bypass:*** Pigs in the CP/CPB group had pronounced myocardial stunning, with ~40–50% decreases in peak systolic LV pressure (LVSP), LV developed pressure (LVDevP), and maximum positive pressure derivative (+dP/dt) (Figure 1A–C) compared to sham animals. The inclusion of rottlerin (500 nM) in the cardioplegia solution (CP/CPB + Rott) significantly improved functional recovery post CP/CPB in all of these parameters (Figure 1A–C). Cardiac relaxation parameters were also altered in the CP/CPB group, with significantly decreased −dP/dt (Figure 1D) and increased Tau (Figure 1E) versus sham, which showed strong trends for normalization in the CP/CPB + Rott groups. Finally, HR was significantly increased in both CP/CPB and CP/CPB + rottlerin-treated groups. Baseline non-normalized values are presented in Table 1.

**Figure 2:**
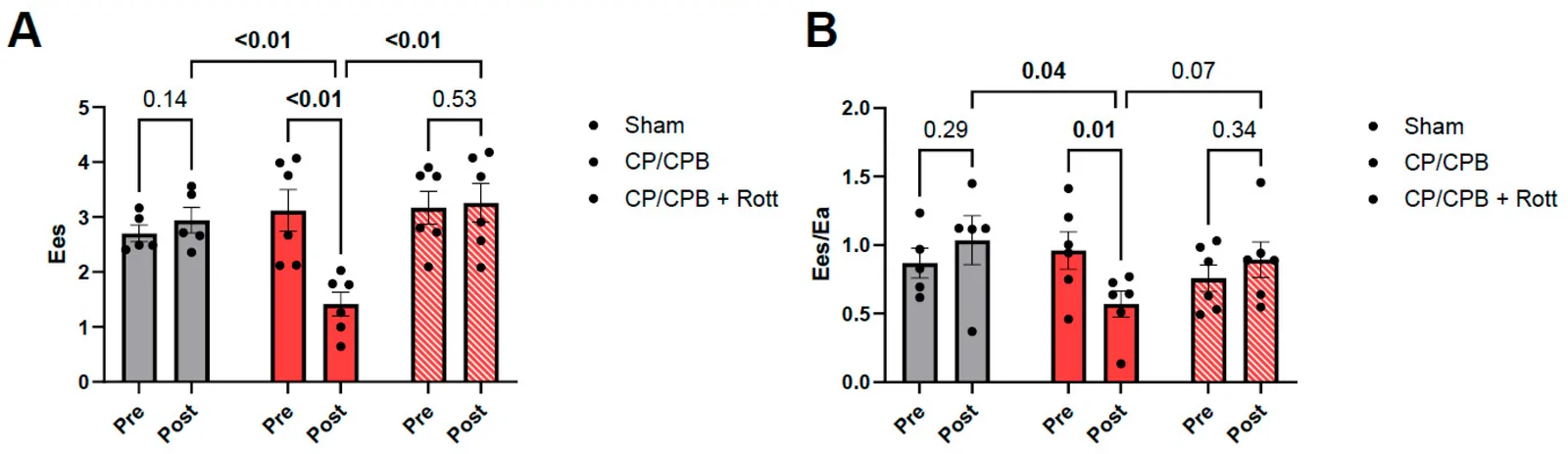
***Rottlerin improves cardiac contractility and ventricular/arterial coupling post CP/CPB.*** (**A**) End-systolic elastance (Ees) and (**B**) Ees/arterial elastance ratio (Ees/Ea). Sham n = 5, CP/CPB n = 7, and CP/CPB + Rott n = 6. *p* as indicated; two-way ANOVA, Holm–Sidak.

Cardiac 500 nM rottlerin (Figure 2A). This increase in contractility was associated with a significant improvement in ventriculoarterial coupling, as shown by the Ees/arterial elastance (Ea) ratio in pigs with cardioplegia supplemented with 500 nM rottlerin (Figure 2B). There were no significant changes in Ea alone.

**Figure 3:**
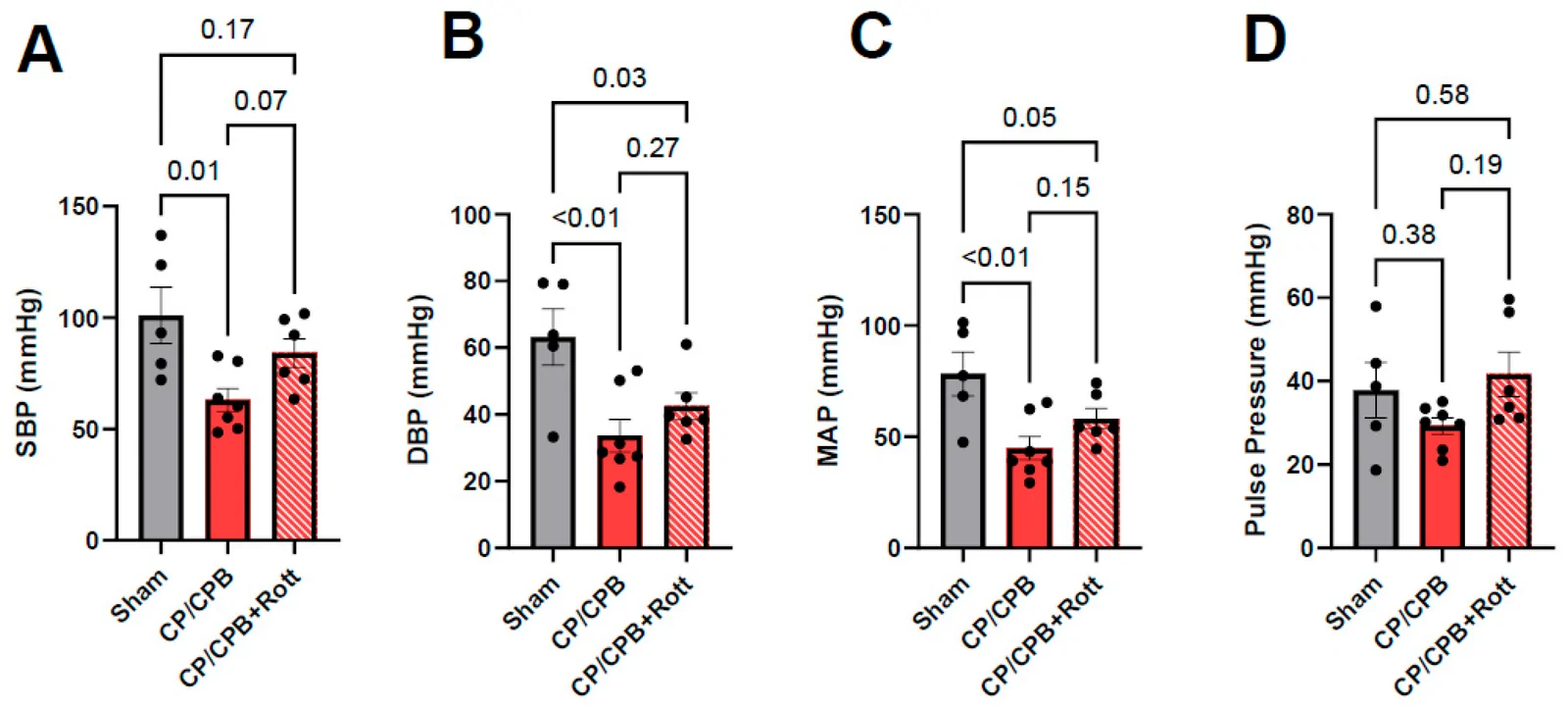
***Rottlerin improves systemic blood pressure post CP/CPB.*** Arterial pressure readings including (**A**) systolic blood pressure (SBP), (**B**) diastolic blood pressure (DBP), (**C**) mean arterial pressure (MAP), and (**D**) pulse pressure (PP). Data are expressed as % change from baseline pre-bypass values. Sham n = 5, CP/CPB n = 7, and CP/CPB + Rott n = 6. *p* as indicated; one-way ANOVA with SNK.

As BK_Ca_ channel activation and rottlerin treatment is associated with robust vascular smooth muscle vasodilation [21,27], we measured arterial pressures at baseline and post bypass. As anticipated, there were significant decreases in systolic (SBP), diastolic (DBP) and mean arterial pressure (MAP) from pre to post CP/CPB treatment (Figure 3A–C). BK_Ca_ activation in the CP/CPB + Rott group showed non-significant trends in improvement versus CP/CPB alone for SBP, DBP and MAP. There were no significant changes in pulse pressure, but trends of a reduction in CP/CPB that was normalized by rottlerin-supplemented CP treatment were observed (Figure 3D). These data indicate that rottlerin as a cardioplegia additive delivered intermittently directly to the arrested coronary circulation does not induce significant systemic vasodilation post bypass.

**Figure 4:**
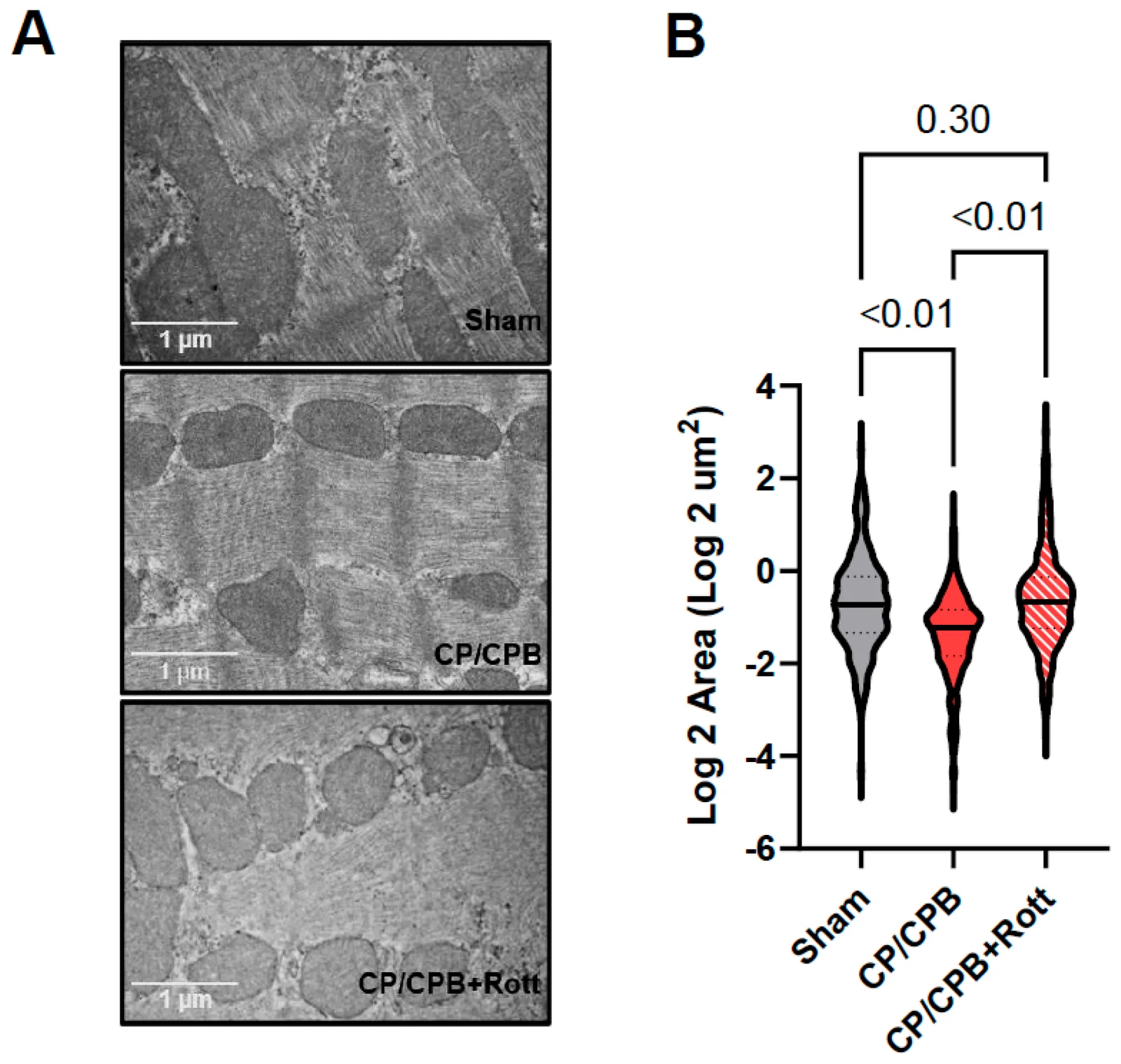
***BK_Ca_ activation increases mitochondrial area post-CP/CPB.*** (**A**) Representative TEM images of LV myocardium 1 h post CP/CPB. Note: images are zoomed and cropped from original images to show mitochondrial detail; original images were acquired at ~25,000x. (**B**) Quantitation of mitochondrial area shown as log2-transformed data. n = 3 sham, 4 CP/CPB and 3 CP/CPB + Rott. Quantitation performed on at least 5 images per tissue with ~50–100 mitochondria per animal. One-way ANOVA with SNK, solid line median, dashed lines upper and lower quartiles.

***Rottlerin increases mitochondrial size, promotes respiratory supercomplexes, and reduces ROS-dependent damage.*** To further evaluate the mechanism of BK_Ca_ activator-induced cardioprotection and increases in contractility, we interrogated potential BK_Ca_-dependent effects on mitochondrial function including mitochondrial volume and electron transport chain (ETC) expression and organization. Electron microscopy of LV tissues demonstrated that CP/CPB decreased mitochondrial area compared to sham treatment (Figure 4A,B). Rottlerin increased mitochondrial area consistent with BK_Ca_-dependent K^+^ flux to increase mitochondrial matrix volume (Figure 4A,B).

**Figure 5:**
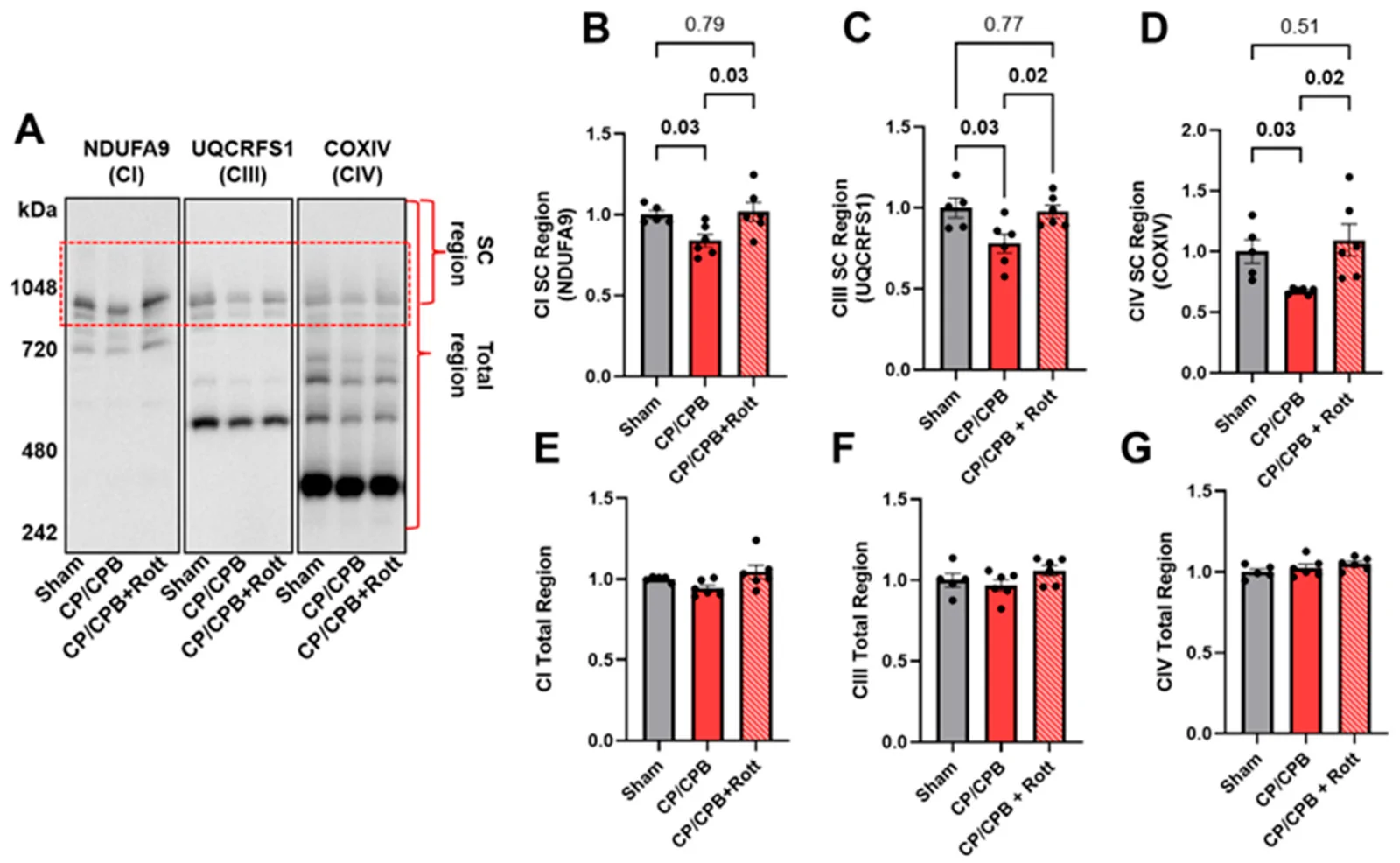
***BK_Ca_ activation increases mitochondrial supercomplex formation post-CP/CPB and lowers ROS-dependent protein modification.*** (**A**) Representative BN-PAGE blots of CI, CIII and CIV in LV myocardium 60 min post CP/CPB. Dashed red box highlights supercomplex region. Quantitation of (**B**) CI, (**C**) CIII, and (**D**) CIV present in the supercomplex region of the gels in (**A**). (**E**–**G**) Quantitation of total complex assembly for CI, CIII, and CIV, respectively (supercomplexes + individual complexes). All densitometry normalized to total mitochondrial protein loaded on the gel. Sham n = 5, CP/CPB n = 7, and CP/CPB + Rott n = 6. *p* as indicated; one-way ANOVA with SNK.

We next examined changes in electron transport chain supercomplexes using BN-PAGE of isolated mitochondria (Figure 5A). We found that CP/CPB significantly decreased mitochondrial supercomplex formation vs. sham treatment (Figure 5B–D), which was normalized in the CP/CPB + Rott groups. There were no changes in total complex assembly (supercomplexes and smaller complexes) including individual and smaller sub-assemblies of complexes I, III, or IV (Figure 5E–G). All data were normalized to total mitochondrial protein load as assessed by far-red imaging of Coomassie-stained BN-PAGE gels.

**Figure 6:**
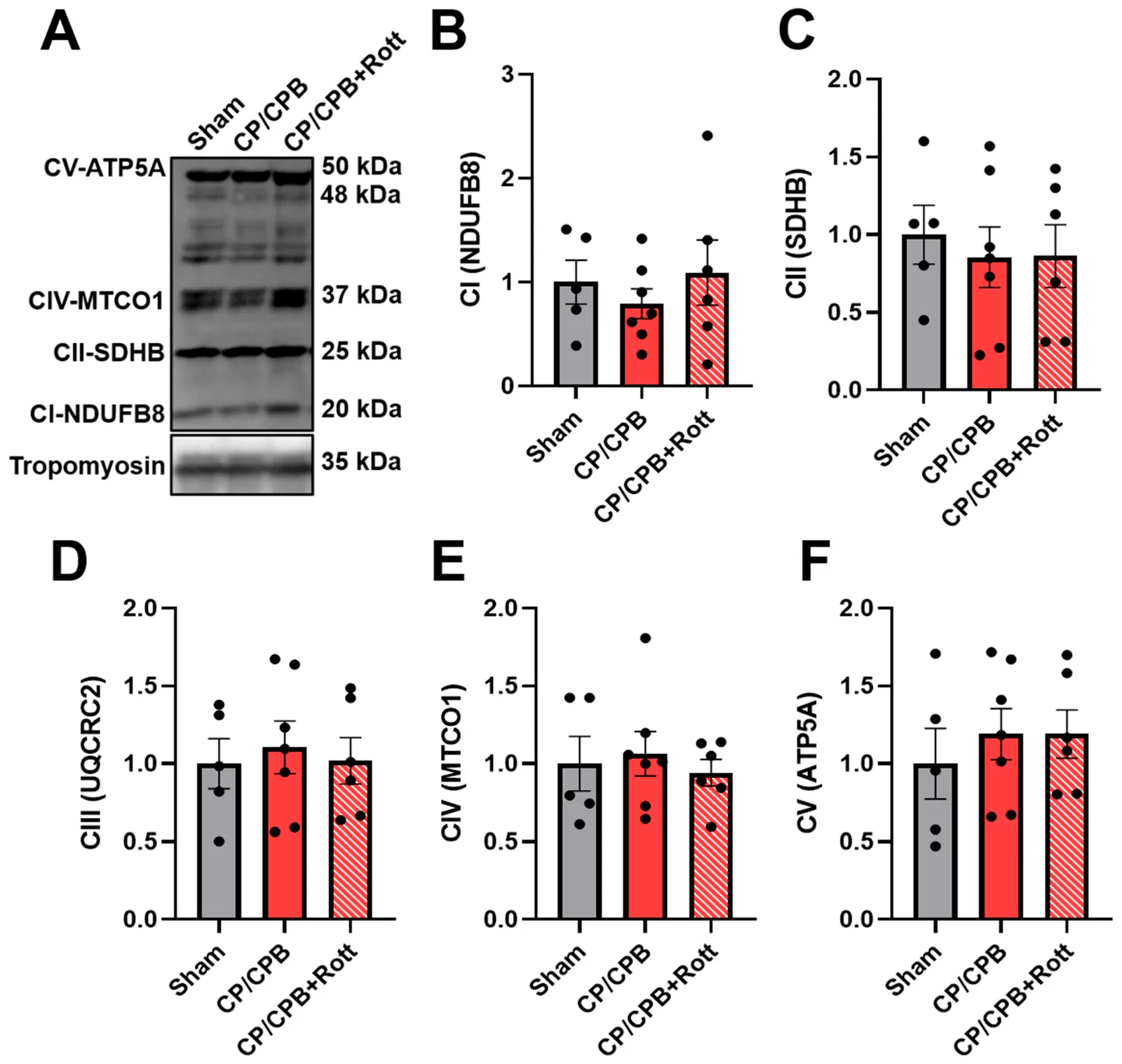
***Neither CP/CPB nor rottlerin changes mitochondrial ETC protein expression.*** (**A**) Representative western blot of individual ETC subunits with an OXPHOS antibody cocktail. (**B**–**F**) Quantitation of results in A for NDUFB8 (CI), SDHB (CII), UQCRC2 (CIII), MTCO1 (CIV), and ATP5A (CV). Sham n = 5, CP/CPB n = 7, and CP/CPB + Rott n = 6. No significant changes; one-way ANOVA with SNK.

There were no changes in the expression of representative subunits of mitochondrial ETC complexes, indicating that there were no gross changes in mitochondrial protein expression in this acute myocardial stunning model (Figure 6A–F).

**Figure 7:**
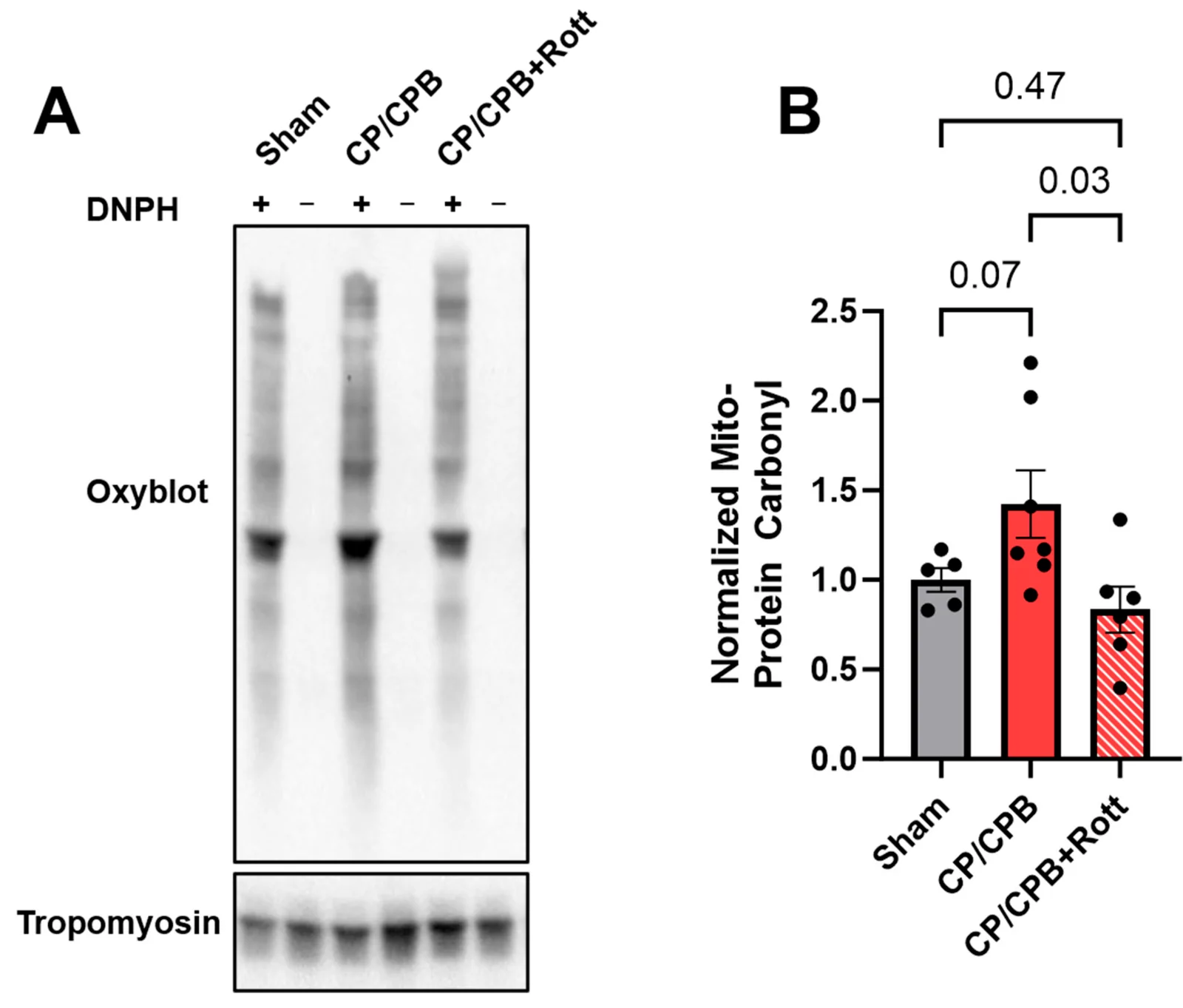
***Rottlerin decreases CP/CPB-induced mitochondrial protein oxidation.*** (**A**) Representative blot of oxidatively modified whole lysate protein. (**B**) Quantification of gel in A corrected for background densitometry of DTT-labeled sample and protein load. Sham n = 5, CP/CPB n = 7, and CP/CPB + Rott n = 6. *p* as indicated; one-way ANOVA with SNK.

***Rottlerin decreases mitochondrial protein oxidation pos-CP/CPB.*** Isolated mitochondrial protein fractions assessed for ROS-dependent protein carbonyl modification showed a strong trend for increased ROS-dependent damage in the CP/CPB group, which was significantly improved in the CP/CPB + Rott group (Figure 7A,B).

**Figure 8:**
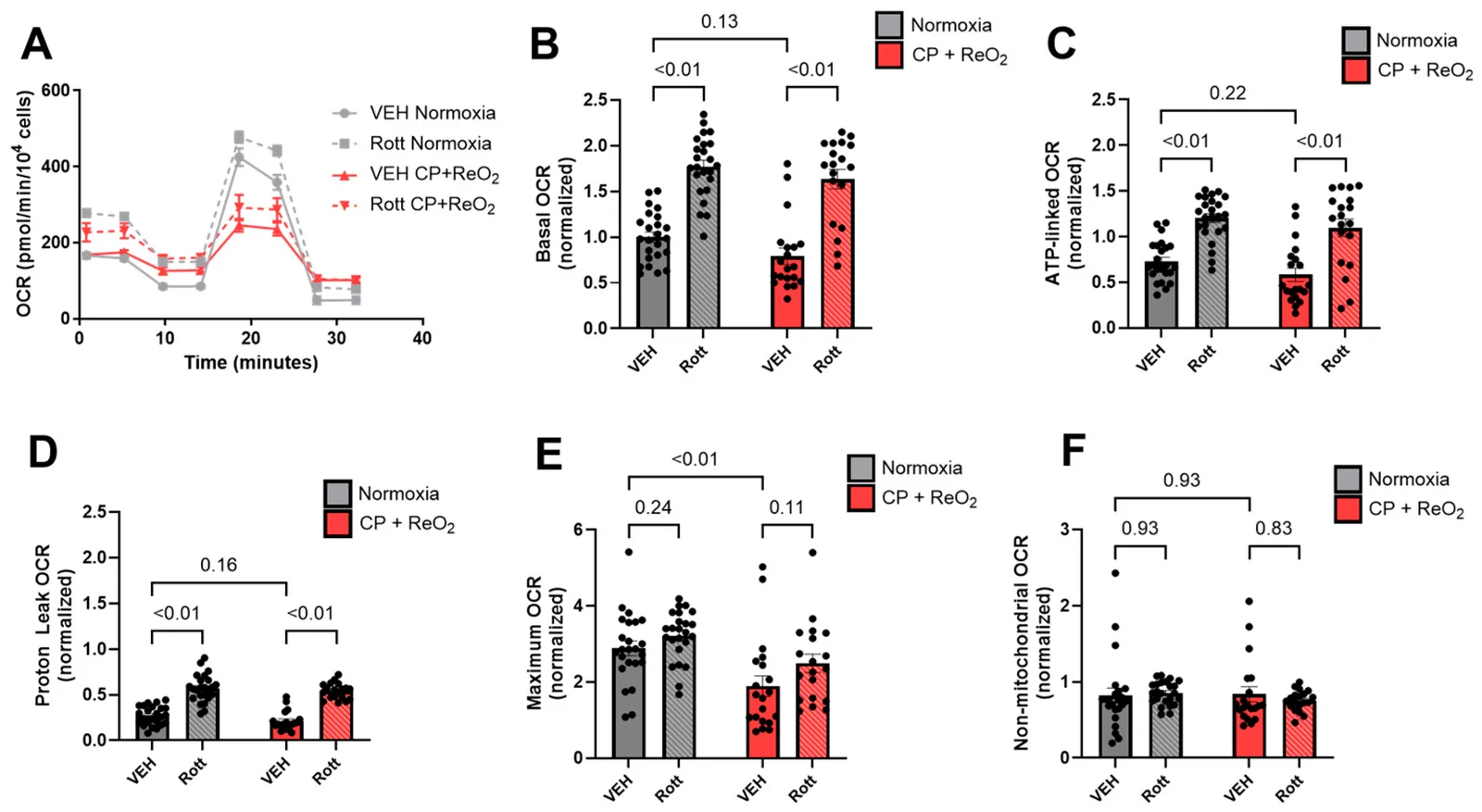
***In vitro CP/reoxygenation decreases respiration that is improved by rottlerin.*** (**A**) Representative seahorse mitostress experiment of normoxic and hypoxic CP/ReO_2_ with and without 500 nM rottlerin. Quantitation of 4 independent experiments showing (**B**) basal, (**C**) ATP-linked, (**D**) proton-leak, (**E**) maximal (FCCP), and (**F**) non-mitochondrial O_2_ consumption. Minimum n = 19 from 4 independent experiments. *p* as indicated; two-way ANOVA with Holm–Sidak. Solid bars represent Normoxia (grey) and CP+ReO_2_ (red). Hatched bars indicate the addition of rottlerin.

***Rottlerin increases mitochondrial respiration in vitro:*** To determine if BK_Ca_ activation increased mitochondrial respiration, we employed an in vitro cold hypoxic cardioplegic arrest model and assessed respiration via a seahorse mitostress assay (Figure 8A). CP/ReO_2_ decreased basal and ATP-linked respiration, which was significantly increased with rottlerin-supplemented CP solution (Figure 8B,C). Rottlerin also increased basal proton leak in both control and CP/ReO_2_-treated cells (Figure 8D). CP/CPB caused pronounced decreases in maximal mitochondrial respiration induced by FCCP. Rottlerin increased maximal respiration, but this was not significant (Figure 8E). Finally, rottlerin did not affect non-mitochondrial oxygen consumption in either control or CP/ReO_2_ cells (Figure 8F). Thus, rottlerin-induced improvements in mitochondrial supercomplex assembly and cardiac function are associated with increased basal and ATP-linked mitochondrial respiration following hypoxic cardioplegia-induced myocardial stunning.

**Figure 9:**
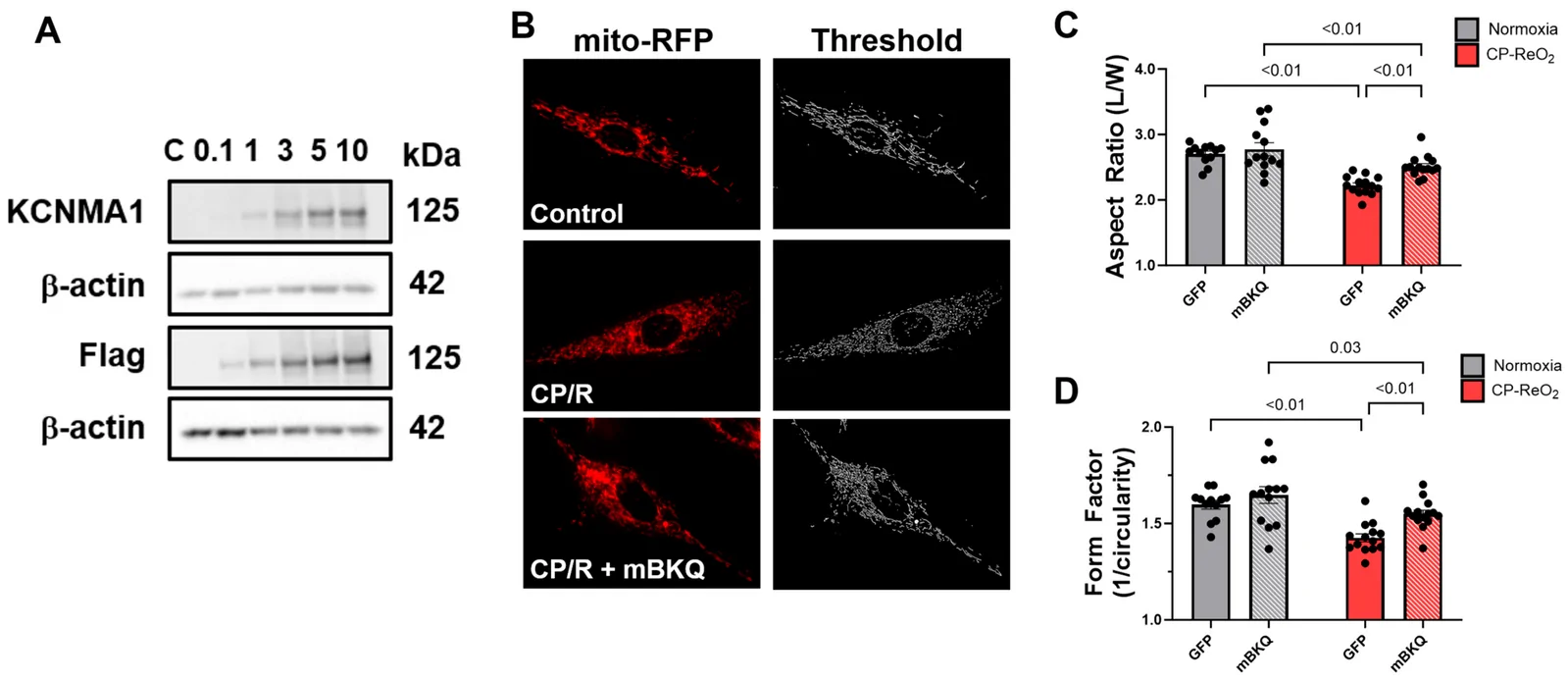
***Mitochondrial BK_Ca_ overexpression modulates mitochondrial morphology.*** In vitro CP/ReO_2_ promotes shorter, less tubular mitochondria which are normalized by mBKQ overexpression. (**A**) Dose–response blot of 48 h adenoviral overexpression of mBKQ (flag-tagged) in H9c2 cells. (**B**) Representative mito-RFP H9c2 cells and corresponding threshold images used for morphometry analysis. (**C**) Quantitation of form factor (1/circularity) and (**D**) aspect ratio (L/W). n = 12–14. *p* as indicated; two-way ANOVA with Holm–Sidak.

***Overexpression of a gain-of-function, mitochondrial-specific BK_Ca_ channel (mBKQ) modulates mitochondrial morphology.*** To support that rottlerin’s effects are mediated via BK_Ca_ channels, we employed adenoviral overexpression of a mitochondrial-specific rat BK_Ca_-DEC splice variant with a gain-of-function mutation R267Q (rat numbering to the analogous R207Q mutation previously described in mice [28] which we called mBKQ. Expression data following 48 h of infection are shown in Figure 9A. CP/ReO_2_ in H9c2 cells stably expressing mito-RFP caused a reduced aspect ratio and form factor, indicating smaller, less tubular mitochondria (Figure 9B–D). mBKQ overexpression significantly increased both aspect ratio and form factor, consistent with rottlerin’s effects on pig LV mitochondria.

**Figure 10:**
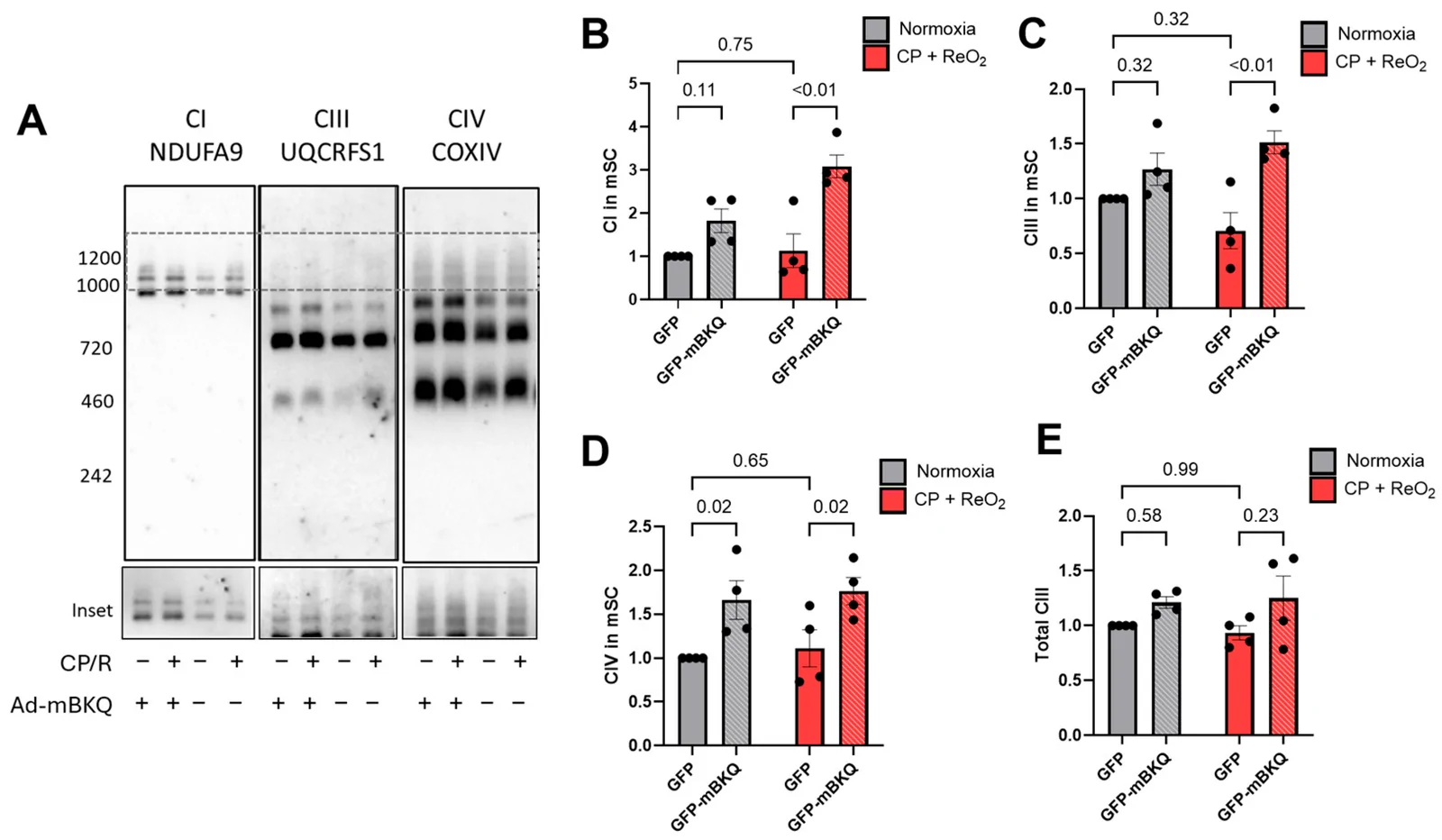
***mBKQ overexpression promotes supercomplex formation.*** (**A**) Representative BN-PAGE of digitonin-solubilized isolated mitochondria with NDUFA9 (CI), UQCRFS1 (CIII) and COXIV (CIV). Inset: darker exposure of boxed region in (**A**). (**B**) Quantitation of CI in mSC region, (**C**) Quantitation of CIII in mSC region, and (**D**) Quantitation of CIV in mSC region, normalized to total CIII signal. (**E**) Quantitation of total CIII (mSC and smaller assemblies). n = 4. *p* as indicated; two-way ANOVA with Holm–Sidak.

***mBKQ expression increases mSCs following in vitro CP/ReO_2_*.** Similar to BK_Ca_ pharmacological activation with rottlerin, mBKQ increased mitochondrial supercomplex formation in both control and CP/ReO_2_-treated H9c2 cells (Figure 10A). There were significant increases in CI, CIII, and CIV incorporation into mSCs (Figure 10B–D), but no changes in total complex III levels, which were used to normalize total protein load (Figure 10E). However, in vitro CP/ReO_2_ did not cause significant decreases in mitochondrial supercomplexes unlike the in vivo pig model, suggesting less severe mitochondrial restructuring in the in vitro cell model.

**Figure 11:**
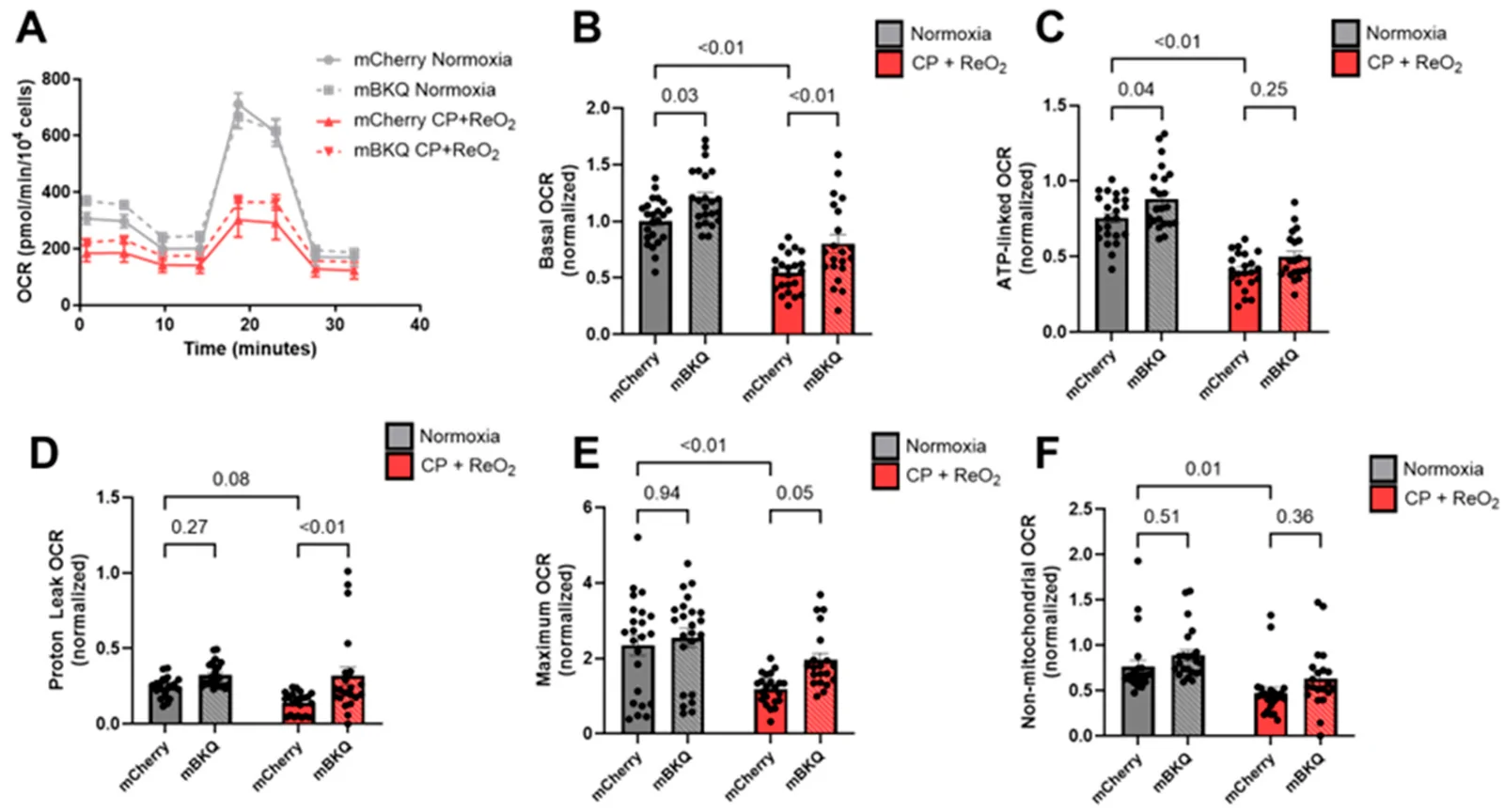
***Gain of Function BK_Ca_ overexpression increases respiration.*** (**A**) Representative seahorse mitostress experiment of normoxic and hypoxic CP/ReO_2_ with and without mBKQ overexpression. Quantitation of 4 independent experiments showing (**B**) basal, (**C**) ATP-linked, (**D**) proton leak, (**E**) maximal OCR (FCCP), and (**F**) non-mitochondrial O_2_ consumption. Minimum n ≥ 20 from 4 independent experiments. *p* as indicated; two-way ANOVA with Holm–Sidak.

***mBKQ increases mitochondrial respiration post-CP/ReO_2_***. In vitro CP/ReO_2_ significantly decreased mitochondrial respiration, as shown by seahorse mitostress analysis (Figure 11A). Adenoviral overexpression of mBKQ caused a significant increase in basal (Figure 11B) and ATP-linked respiration (Figure 11C), as well as proton leak (Figure 11D), similar to rottlerin (Figure 8). Interestingly, mBKQ overexpression resulted in increased maximal respiration in these cells (Figure 11E), which was not observed with pharmacologic manipulation of BK_Ca_ activation with rottlerin. This difference may suggest that overexpression of the channel for 48 h may result in additional mitochondrial changes compared to acute activation.

**Figure 12:**
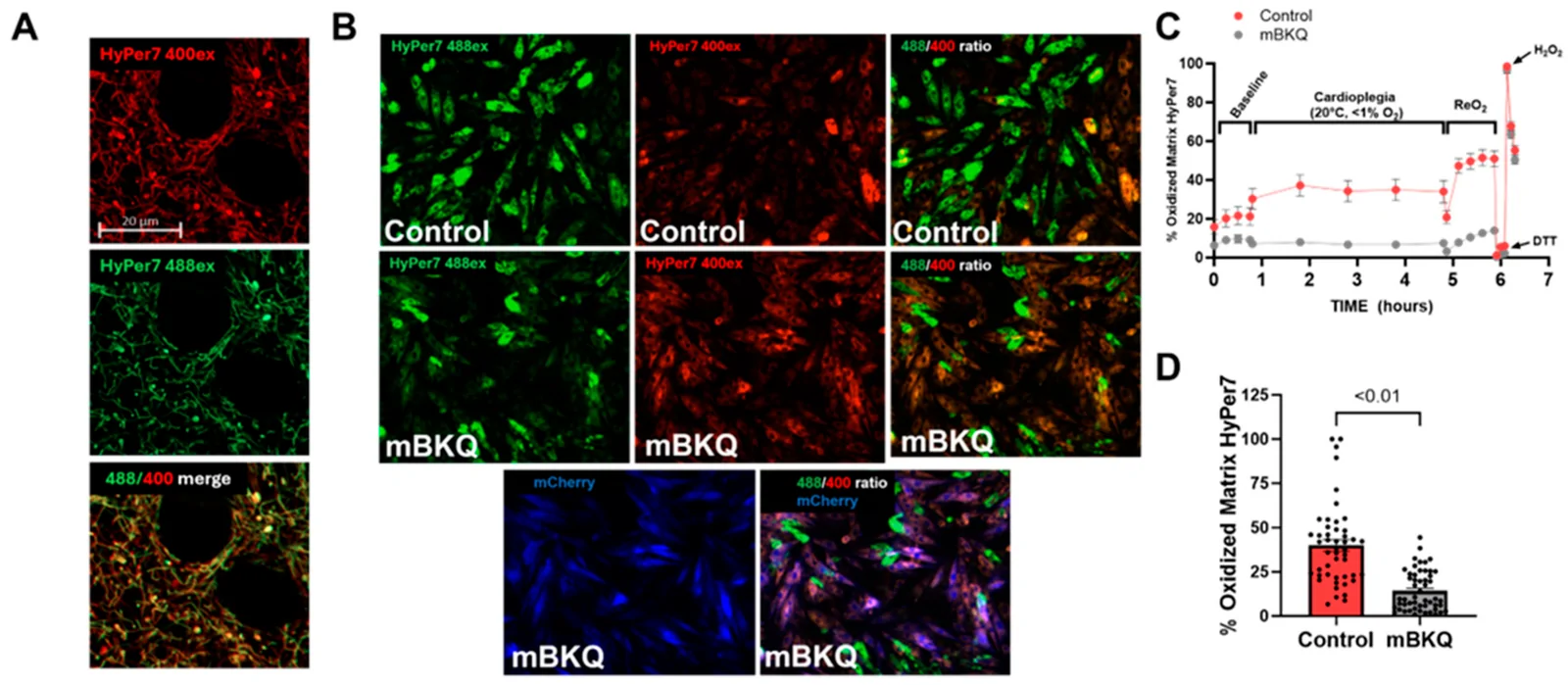
***mBKQ decreases ROS generation following CP/ReO_2_.*** (**A**) High-resolution/magnification representative images demonstrating mitochondrial localization of HyPer7 in H9c2 cells. (**B**) Representative images of control and mBKQ-infected cells at the end of CP/ReO_2_. Psuedocolored images: Green: HyPer7 with 400 nm excitation, Red: HyPer7 with 400 nm excitation Blue: mcherry fluorescence. (**C**) Quantitation of HyPer7 488/400 ration during baseline, CP, ReO_2_ and DTT (5 mM) and H_2_O_2_ (600 mM). (**D**) Quantitation of final ReO_2_ timepoint in **C**. n = 48–52 wells from 4 independent experiments. *p* as indicated.

***BK_Ca_ overexpression reduces mitochondrial ROS.*** We also examined mitochondrial ROS generation in our in vitro CP/ReO_2_ model using adenoviral overexpression of the mitochondrial matrix-targeted oxidant sensor HyPer7. matrix-HyPer7 is a ratiometric ROS sensor that upon oxidation displays increased fluorescence when excited at 488 nm and decreased fluorescence when excited at 400 nm [26]. The signal was normalized to minimum (DTT treatment, 0%) and maximum oxidation (H_2_O_2_, 100%) at the end of the experiment. Figure 12A shows mitochondrial localization of matrix-Hyper7 excited at 488 nm and 400 nm in a high-resolution micrograph. Representative 20× images from our plate-based imaging assay at 1 h reoxygenation are shown in Figure 12B. CP/ReO_2_ caused significant increases in mitochondrial ROS generation, which was significantly reduced with Adv-mBKQ (Figure 12C,D).

## 4. Discussion

In this manuscript, we demonstrate that the BK_Ca_ activator rottlerin is a potent cardioprotective agent that limits myocardial stunning and improves functional recovery in a preclinical, translationally relevant large animal model of CP/CPB. To our knowledge, this is the first large animal study evaluating the cardioprotective effect of BK_Ca_ channels in an in vivo ischemic model. The BK_Ca_ activator rottlerin as a supplement to cardioplegia solution robustly improved cardiac contractility upon weaning and reperfusion following CP/CPB. This cardiac functional improvement was associated with normalization of mitochondrial volume as shown by TEM, reductions in mitochondrial protein oxidative modifications, and a significant increase in the assembly of mitochondrial ETC supercomplexes (mSCs). In vitro CP and reoxygenation resulted in depressed mitochondrial respiration, which was normalized with rottlerin-dependent BK_Ca_ activation and overexpression of gain-of-function mutant mito-BK_Ca_ channels. Furthermore, adenoviral overexpression of BK_Ca_ channels in cells was generally sufficient to mimic the in vivo rottlerin effects, modulate mitochondrial morphology, improve respiration, increase mSC assembly, and reduce ROS generation.

Rottlerin is an under-recognized BK_Ca_ channel activator. In the channel literature, rottlerin is primarily known as mallotoxin. Rottlerin/mallotoxin activates BK_Ca_ channels in the nM range and can shift voltage-dependent activation of the channel −70 mV [21,22,29]. This makes rottlerin one of the most potent BK_Ca_ activators identified. Interestingly, rottlerin’s effects are similar to specific BK_Ca_ gamma subunits (LRRC26, 38, 52, and 55, BKγ1-4), and rottlerin is ineffective in BK_Ca_ channels with LLRC26 and other specific BKγ-subunits [22,29,30,31], suggesting that rottlerin interacts with the pore-forming subunit by mimicking gamma subunit interactions. Additional studies in our lab could not document LRRC26 or other γ-subunit expression in mitochondria using DIA-MS or Western blot (unpublished observations. Our previous studies in rodents have confirmed that at the low doses employed (0.1–1 μM), rottlerin’s cardioprotective effects are mainly attributable to BK_Ca_ activation, as shown by inhibition of cardioprotection with paxilline in rats and a complete lack of cardioprotection in BK_Ca_ KO mice [17,18].

Based on our data, we propose that a key mechanism of BK_Ca_-dependent cardioprotection is the promotion of mitochondrial supercomplexes as a key factor to limit ROS generation and improve respiration. Both reduced ROS and increased respiration have long been recognized as important factors for BK_Ca_-dependent cardioprotection, but how K^+^ flux modulates both has been unclear. Increased mSCs likely promote more efficient respiration with less electron slippage and ROS development [32]. Indeed, we find that rottlerin completely normalizes CP-induced mSC degradation in pigs, which is associated with significantly less mitochondrial protein ROS-dependent modification in vivo. Rottlerin also potently increased respiration in vitro. In addition, we previously showed that rottlerin preserves ΔΨm in a similar model of CP/ReO_2_, indicating that this increased respiration is not due to extensive uncoupling at the doses employed [17]. Adenoviral overexpression of gain-of-function mBKQ caused significant increases in mSC formation both in control and CP/ReO_2_ cells. mBKQ-infected cells did not have as robust increases in respiration as rottlerin; however, respiration was elevated and there was significantly less mitochondrial-specific ROS generation in these cells, suggesting more efficient respiration. In support of BK_Ca_-induced mSC mediating cardioprotection in response to ischemia, increased K^+^ flux due to activation of small conductance K^+^ channels can promote mSC assembly in a mouse model of catacholeminergic ventricular tachycardia, which is associated with reduced ROS [33]. Therefore, the data support that rottlerin-induced BK_Ca_ activation drives mSC assembly, suggesting a mechanism for reduced ROS and improved respiration.

As noted above, there were minor differences in respiration between pharmacological and mBKQ overexpression concerning respiration. The timing of pharmacological activation (3 h) and the timing of overexpression of Adv-mBKQ (48 h) are extremely different, which may explain some minor discrepancies in respiration and mSC assembly. In addition, differences in respiration between rottlerin and mBKQ may reflect differing sensitivity to negative feedback respiratory control mechanisms, which we anticipate are more likely to remain intact with overexpression of BK_Ca_ channels compared to robust pharmacologic activation which may bypass these feedback control mechanisms. Indeed, BK_Ca_ channels have robust negative feedback regulation from electron transport chain substrates including NADH, succinate, and glutamate [34], as well as voltage-dependent inactivation associated with high ΔΨm, suggesting that BK_Ca_ channel opening may be a significant positive regulator of respiration during mitochondrial stress.

The precise mechanism of BK_Ca_-dependent promotion of mitochondrial supercomplexes is currently unclear. Our hypothesis is that increases in K^+^ flux into the mitochondria normalize mitochondrial volume following mild I/R injury. Note that we suspect volume changes in CP/reoxygenation due to the reduced driving force for K^+^ flux associated with decreased ΔΨm occurring in the absence of mPTP opening (which would induce significant swelling alone) [13]. We propose that BK_Ca_-induced increases and/or normalization in volume necessitates mitochondrial structural and membrane remodeling, including increases in mitochondrial size and cristae formation. This alteration in mitochondrial structure likely provides more favorable locations for the assembly of mitochondrial supercomplexes. It is likely that this will involve specific cristae and/or membrane-modifying proteins involved in the synthesis or processing of membrane lipids such as cardiolipin, as well as proteins associated with enhanced cristae formation including prohibitins 1 and 2, Opa1, and members of the mitochondrial cristae organizing system (MICOS), among others [35,36,37,38]. Alternative hypotheses regarding BK_Ca_-dependent promotion of mSCs may be associated with the observed decrease in mitochondrial ROS, as ROS may negatively impact mSC formation or maintenance; thus, if BK_Ca_-reduced ROS are upstream of mSC formation, they may in fact serve to preserve mSC structure. Future studies employing KO and overexpression approaches of mSCs and cristae-modifying proteins will be needed to definitively determine their involvement in cardioprotective mitochondrial functional changes downstream of BK_Ca_. Linking BK_Ca_-dependent or other K^+^ channel-dependent volume changes to cristae structural modifications and subsequent mSC assembly is a much needed area of future investigation. Furthermore, studies employing knockdown of mSC assembly factors such as Cox7a2l, HIGD proteins, and NDUFAF4 [39] will be needed to determine if BK_Ca_-induced preservation of mSCs is required for BK_Ca_-induced decreased ROS and improved respiration in CP/CPB.

The data presented in this manuscript and our previous studies support that the beneficial effects of rottlerin, at the doses employed, are attributable to BK_Ca_ channels. This is based on current and previous studies showing the following: (1) cardioprotection in rat hearts is inhibitable by the BK_Ca_ blocker paxilline [18], (2) rottlerin shows no cardioprotective activity in BK_Ca_ KO mouse hearts [17], and (3) adenoviral overexpression of mitochondrial BK_Ca_ channels can mimic many of the effects of rottlerin. However, despite being a highly potent BK_Ca_ activator, we should point out that rottlerin has been attributed numerous activities. Rottlerin has been described previously as a direct oxygen radical scavenger, but this activity is quite weak in DPPH scavenging assays (IC50 > 100 µM) [40] and unlikely to play a role in our model considering the concentrations (500 nM) employed in this study. Rottlerin is also often identified as a PKCδ inhibitor; however, any direct effects of rottlerin on PKCδ are unlikely or are associated with much higher potentially non-specific doses than those needed for BK_Ca_ activation, usually in the 5–10 μM range [41]. Rottlerin at higher doses is recognized as a mitochondrial uncoupler. Many of the kinase-related effects of rottlerin, including PKCδ inhibition and AMPK activation, as well as cytotoxic effects in many cell lines, could be attributed to reductions in ATP that are likely associated with excessive uncoupling activity [42]. It is possible that these effects may be an indirect result of inappropriate and excessive BK_Ca_ activation, as BK_Ca_ activation is anticipated to reduce ΔΨm and the inner membrane proton gradient through robust K^+^/H^+^ exchange [13,42]. Importantly, in our previous studies, we showed that at doses less than 1 μM in our CP/reoxygenation model, rottlerin does not diminish ΔΨm [17], but rather increases it following 30 min of reoxygenation. When rottlerin is given acutely (500 nM), we do not observe any significant decreases in ΔΨm in isolated rat myocytes (unpublished observations). We hypothesize that the lack of membrane potential changes is influenced by two factors in our model: (1) at the doses employed, significant appropriate negative feedback regulation of the BK_Ca_ channel remains intact, and (2) we only include rottlerin in the CP solution, delivered intravascularly and intermittently (every 20 min) and removed upon reperfusion, thereby limiting potential uncoupling activity. Overall, our current data support that rottlerin’s cardioprotective effects in this model are most likely attributable to BK_Ca_ activation, but the possibility of additional effects of rottlerin contributing to cardioprotection cannot be ignored.

There are a number of factors limiting the translational potential of rottlerin as a BK_Ca_ activator in the clinic. First, there are numerous effects attributed to the molecule in the μM range, thus defining an extremely narrow therapeutic window. However, in our CP/CPB pig and previous rodent isolated heart studies, rottlerin was directly delivered to a static coronary circulation, where its major effects including potential coronary vasodilation and cardiomyocyte mSC formation are beneficial, potentially demonstrating clinical utility in this highly specialized situation. However, BK_Ca_ channels are ubiquitously expressed, influencing numerous endocrine, smooth muscle and neuronal processes; thus, off-target effects due to BK_Ca_ activation in other organs will likely be problematic for systemic delivery. Harnessing the cardioprotective benefits of mitochondrial BK_Ca_ channels for other applications will likely require targeted gene therapy strategies and/or compounds with improved specificity for mitochondrial BK_Ca_ channels.

## 5. Conclusions

In summary, we demonstrate that BK_Ca_ activation with the small molecule rottlerin robustly improves cardiac contractility in a large animal model of myocardial stunning associated with cardioplegic arrest and cardiopulmonary bypass. This was associated with the normalization of mitochondrial volume, the preservation of mitochondrial supercomplexes, and reduced ROS in vivo. Both BK_Ca_ activation with rottlerin and/or gain-of-function mitochondrial-specific BK_Ca_ channel overexpression increased respiration, increased mSCs and reduced ROS. These results suggest that BK_Ca_ activation could be a potent cardioprotective therapeutic strategy for ischemic insults associated with cardiac surgery or transplant.

## Figures and Tables

**Table 1:** Baseline hemodynamic parameters: LVSP: left ventricular systolic pressure; LVDevP: left ventricular developed pressure; SBP: systolic blood pressure; DBP: diastolic blood pressure; MAP: mean arterial pressure; Ees: end-systolic elastance. *p* as shown; one-way ANOVA with SNK vs. sham.

Hemodynamic Parameters	Sham		CP/CPB			CP/CPB + Rott		
	Mean	S.D.	Mean	S.D.	*p*	Mean	S.D.	*p*
LVSP (mmHg)	100.04	20.20	120.53	16.50	0.13	110.69	11.33	0.34
LVDevP (mmHg)	99.07	21.04	113.81	18.99	0.83	101.24	12.05	0.86
+dP/dt (mmHg/s)	1545.40	418.85	1517.97	473.02	0.92	1297.00	274.23	0.60
−dP/dt (mmHg/s)	−2238.80	722.19	−2574.85	890.07	0.73	−2060.50	448.89	0.73
Tau (ms)	42.84	9.18	48.94	10.75	0.38	51.44	9.62	0.38
Heart rate (bpm)	75.46	19.79	73.40	14.82	0.94	78.44	6.42	0.94
SBP(mmHg)	102.12	19.07	119.55	17.44	0.25	105.88	14.86	0.75
DBP (mmHg)	67.36	10.92	77.88	15.40	0.38	67.17	9.78	0.98
MAP (mmHg)	82.03	14.33	95.99	15.43	0.27	83.81	12.50	0.85
Ees	2.70	0.30	3.12	0.92	0.55	3.17	0.73	0.48

LVSP: left ventricular systolic pressure; LVDevP: left ventricular developed pressure; SBP: systolic blood pressure; DBP: diastolic blood pressure; MAP: mean arterial pressure; Ees: end-systolic elastance. *p* < 0.05, One Way ANOVA, Dunnet’s test vs. Sham.

## Data Availability

No new data were created or analyzed in this study. Data sharing is not applicable to this article.

## Abbreviations

The following abbreviations are used in this manuscript:

BK_Ca_	Large conductance Ca^2+^-activated K^+^ channels
CABG	Coronary Artery Bypass Graft
CP/CPB	Cardioplegic arrest and cardiopulmonary bypass
MI	Myocardial infarction
CP/ReO_2_	CP/reoxygenation;
mSC	Mitochondrial supercomplex
ETC	Electron transport chain
ROS	Reactive oxygen species
BN-PAGE	Blue native polyacrylamide gel electrophoresis
DTT	Dithiothreitol
Ees	End-systolic elastance
LVSP	Left ventricular systolic pressure
LVDevP	Left ventricular developed pressure
SBP	Systolic blood pressure
DBP	Diastolic blood pressure
MAP	Mean arterial pressure
DNPH	2,4-dinitrophenylhydrazine
FF	Form factor
AR	Aspect ratio
